# Intranasal esketamine significantly alleviates depression severity and suicidal ideations in electroconvulsive therapy (ECT) non-responders

**DOI:** 10.1007/s00406-025-02041-9

**Published:** 2025-07-01

**Authors:** Erhan Kavakbasi, Kevin Rosemann, Mert Yilmaz, Helmut Berndt, Bernhard T. Baune

**Affiliations:** 1 Department of Psychiatry, University Hospital Münster, University of Münster, Albert-Schweitzer-Campus 1, Building A9, 48149 Münster, Germany; 2 Department of Psychiatry, Melbourne Medical School, The University of Melbourne, Melbourne, Australia; 3 The Florey Institute of Neuroscience and Mental Health, The University of Melbourne, Parkville, VIC Australia; 4 Department of Psychiatry, Psychotherapy and Psychosomatics, RWTH Aachen University, Aachen, Germany

**Keywords:** Treatment resistant depression, Esketamine, Major depressive disorder, Novel antidepressants, Electroconvulsive therapy

## Abstract

**Introduction:**

Intranasal esketamine (ESK) is a novel therapy option in patients with treatment-resistant depression (TRD). Patients with a history of electroconvulsive therapy (ECT) non-response (ECT+) in the current episode have usually been excluded from previous studies. Data on the effectiveness of ESK in ECT non-responders are sparse.

**Methods:**

In this retrospective study, we investigated the effectiveness of intranasal ESK in real-world inpatients with (ECT+, *n* = 39) history of ECT non-response compared to patients who have not received an adequate course of ECT in their current episode (ECT-, *n* = 57). A factorial analysis of variance (ANOVA) has been used to determine the impact of ECT non-response on treatment outcome.

**Results:**

A total of *n* = 96 patients (mean age 47.0; 52.1% women) with TRD were included in this study. There was a significant main effect of history of ECT non-response on MADRS score in the ANOVA (F = 10.386, *p* = 0.002). However, there was no significant interaction effect of time (pre-treatment, post-treatment)*history of ECT non-response in current episode (F = 2.166, *p* = 0.143). The response (34.9% vs. 21.9%, χ2 = 1.498, *p* = 0.167) and remission rates (24.4% vs. 12.1%, χ2 = 1.861, *p* = 0.141) were none significantly lower in the ECT + group than in the ECT- group. There was significant improvement in MADRS and BDI-II in the ECT + group. No major safety concerns occurred during the study.

**Conclusion:**

There was no significant impact of ECT non-response on esketamine treatment outcome. Our results support the approach to offer esketamine to ECT non-responders given that the array of treatment alternatives is limited for these patients.

## Introduction

Major depression is a common mental illness with a lifetime prevalence of about 20% [1]. Approximately 30% of depression patients do not adequately respond to antidepressant pharmacotherapy and are considered treatment-resistant in research settings [2]. In the real-world population up to 55% of patients with major depression are estimated to suffer from treatment-resistant depression (TRD) [2]. The response and remission rates to antidepressant pharmacotherapy decrease with each unsuccessful treatment attempt, whereas there is an increase in the rate of side-effects from trial to trial [3]. The later remission occurs during the course of treatment, the higher the risk of relapse increases [3]. Limitations of existing antidepressant agents urge the need for innovative treatment approaches in the management of major depression. In recent years the anesthetic ketamine as well as its s-enantiomer esketamine are in clinical use in patients with TRD [4]. Intranasal esketamine has been approved in treatment-resistant depression as well as in patients with suidical ideations in combination with an oral antidepressant [4, 5]. The efficacy and safety of esketamine intranasal spray have been investigated in phase 3 randomized, placebo-controlled trials [6, 7]. In patients with TRD defined as non-response to at least two antidepressants, esketamine intranasal spray (56 mg or 84 mg) or placebo have been administered in combination with a newly initiated SSRI (selective serotonin reuptake inhibitor) or SNRI (serotonin norepinephrine reuptake inhibitor) twice weekly [6, 7]. Electroconvulsive therapy (ECT) is another important and well-established treatment option in patients with TRD, which is recommended in international guidelines with Level I efficacy [8]. The response rate to ECT ranges between 58 and 70% in meta-analytic reports, which means that about one third of patients do not respond to ECT [9]. There is an ongoing debate on the superiority of either of the treatment options, ECT and ketamine, in major depression. A randomized controlled-trial from Sweden revealed that ECT was superior over racemic ketamine in terms of remission rates (63% vs. 46%) [10]. Thereafter, another randomized trial suggested a non-inferiority of ketamine in comparison to ECT in patients with nonpsychotic TRD [11]. There were significant differences in the baseline characteristics between the two study populations, which likely contributed to the contradictory results of the two studies and should be considered in further investigations. Head-to-head comparisons of ECT and intranasal esketamine are lacking so far. In clinical practice, patients usually will be offered the other option subsequently, if they fail to respond to the first of the two options, ECT or esketamine. Typically, patients with a history of electroconvulsive therapy (ECT) non-response in the current episode have not been included in the early esketamine intranasal spray clinical trials [7, 12, 13]. Thus, the transferability of these study results in real-world samples is limited. Response and remission rates to esketamine in patients with a history of ECT non-response in the current episode are not known. But simultaneously, patients with ECT non-response in the current episode may seek for innovative treatment options such as esketamine in clinical practice. The clinical management of patients not responding to ECT is challenging, since treatment options are sparse for this clinical constellation. Martinolli et al. have published one of the largest intranasal esketamine real-world samples and reported on considerable response and remission rates in real-world patients, where 35% of patients had a psychiatric comorbidity, 15% of the patients were diagnosed with a personality disorder [14]. Only one patient had a history of ECT, thus there is no conclusion regarding the efficacy of intranasal esketamine in patients with a history of ECT non-response in the current episode [14]. Overall, data from real-world practice including ECT non-responders are sparse. The aim of this retrospective study is to investigate the real-world clinical effectiveness of intranasal esketamine regarding depression severity as well as suicidal ideations in patients with a history of ECT non-response (ECT+) in the current episode in comparison to patients who did not receive an adequate ECT series in their current depressive episode (ECT-). We compared changes in depression severity and suicidal ideations as well as in response and remission rates between these two groups.

## Methods

### Data collection

Institutional review board approval has been obtained from the Ethics Board Münster (2023-435-f-S, September 1th, 2023). As this is a retrospective study and only routine clinical data have been used, written informed consent from the participants was not required due to local regulations. The study was conducted according to the ethical principles involving human subjects as stated in the Declaration of Helsinki. Using the esketamine treatment calendar, we have retrospectively identified all patients with major depressive disorder, who received esketamine intranasal spray in the Department of Psychiatry at the University Hospital Münster, Germany. However, we have excluded patients who had responded to ECT previously and had been switched to esketamine due to ECT side-effects (*n* = 5). The patients’ medical records and files including documentation on medical rounds, treatment summaries, records on physical and psychiatric examinations as well as follow-up investigations and previous medical records have been screened to obtain information on the medical and psychiatric history, physical and psychiatric comorbidities and treatment history as well as course of disease and treatment. ECT treatment history and response status to previous ECT series in the current episode have been assessed during patients’ interview as well as by evaluating previous medical records. We have also obtained results of clinical rating scales performed prior and after the esketamine treatment series in routine clinical practice. Patients with a history of ECT non-response in their current depressive episode (ECT+) have been compared to patients who have not received an adequate course of ECT (ECT-) in the current episode. The vast majority of this group consisted of ECT naïve patients, along with patients who received fewer than 6 sessions of ECT during the current episode. All data have been collected in a SPSS Statistics file (Version 28.0.1.1., IBM, Armonk, NY, USA), which has been used for the statistical analyses.

### Esketamine treatment

The FDA (Food and Drug Administration) approved esketamine nasal spray has been used for esketamine treatment [15]. The indication for esketamine treatment has been determined by a senior psychiatric consultant. All treatments have been initiated in an inpatient treatment setting. Patients provided written informed consent on esketamine treatment prior to initiation of treatment. Potential contraindications such as uncontrolled hypertension or acute coronary heart disease have been ruled out before treatment initiation [16]. All patients have been classified as treatment resistant (TRD, treatment-resistant depression). According to local standards TRD is considered in patients, who have not responded to at least two antidepressant agents in adequate dose and duration in their current depressive episode. The reason and indication to initiate esketamine either was TRD or the presence of an acute psychiatric crisis, which has been classified as a psychiatric emergency by the treating physician. In this sample, all patients with a psychiatric emergency fulfilled simultaneously the criteria for treatment-resistant depression. They deteriorated and developed a psychiatric emergency while being treated due to treatment-resistant depression and then received esketamine for the management of acute crisis. However, most patients have been treated with esketamine for the management of TRD without a psychiatric emergency or crisis.

Patients have been advised not to eat any food within two hours prior to esketamine treatment on treatment days. Blood pressure was measured before esketamine administration, after 10 min and after 40 min. Patients have been observed clinically for further 2 h. Esketamine has been self-administered by the patients under the supervision of a health care professional. Before the first session, patients were trained in the use of the nasal applicator with a dummy. Up to five patients have simultaneously been observed during one treatment sessions in the same treatment room, which was kept darkened and quiet. Esketamine nasal spray is provided in applicators containing 14 mg for each nostril resulting in 28 mg in each applicator. The treatment dose was determined by the treating physician. Initial esketamine dose was 56 mg (2 applicators) in patients with treatment-resistant depression, the dose was usually increased to 84 after the first or second sessions. In psychiatric emergencies patients started with 84 mg (3 applicators) from the very beginning. The esketamine administration usually requires a pause of 5 min between each applicator. Esketamine treatments usually took place on two days a week. After an acute treatment phase of 4 weeks, the physicians had the option to terminate esketamine or to extend the treatment series for further treatment weeks. In case of adverse events, dose reduction was a possible option. Treatment as usual was continued during the esketemine series, which included psychotherapy and adjustment or change in oral medication. Clinical assessment of depression severity has been performed with the investigator-rated Montgomery Asberg Depression Rating Scale (MADRS) [17] as well as with the self-rated Beck’s Depression Inventory II (BDI-II) [18] before the initiation of esketamine treatment as well as after the termination of the esketamine series. For assessment of suicidality, we used the item 10 of the MADRS scale rating suicidal thoughts [17]. Clinical ratings were conducted by the treating psychiatrist or psychologist. The follow-up evaluation took usually place in the first days (up to one week) after the last esketamine treatment session. As the ratings took place in routine clinical practice, the raters were not blinded. Response was defined as an at least 50% reduction in MADRS score, whereas patients with a MADRS score of 10 or less at the end of esketamine treatment were considered as remitters.

### Statistical analyses

To assess the impact of the ECT response status in the current episode on treatment outcome, we utilized a factorial analysis of variance (ANOVA). In this model the MADRS score was the dependent variable, age and number of treatment sessions were covariates and the following variables were independent fixed factors: time (pre-treatment, post-treatment), history of ECT non-response in the current depressive episode, psychiatric comorbidity (presence of at least one psychiatric comorbidity), class of concomitant medication (SSRI, SNRI, other), gender as well as the main indication for esketamine treatment (treatment-resistant depression, psychiatric emergency). As an exploratory analysis, we also used the same model to assess changes in BDI-II score as well as in suicidality. For this reason, we used BDI-II and MADRS item 10 scores as dependent variables in the respective subsequent calculations. To compare response rates to esketamine between previous ECT non-responders (ECT+) and patients who did not receive an adequate course of ECT during the current depressive episode (ECT-) we used the χ2 test.

## Results

### Baseline data

A total of *n* = 96 patients (52.1% women) with major depressive disorder were included in this study. All patients fulfilled the criteria for treatment-resistant depression with at least two unsuccessful antidepressant trials in the current depressive episode. The mean age of patients was 47.0 years (median 48.0, min. 21, max. 79, range 58). Depression with psychotic features was present in 5.2% (*n* = 5). The majority of patients (58.3%, *n* = 56) had at least one comorbid psychiatric disorder in addition to major depression. Personality disorder (*n* = 16, 16.7%), substance use disorder (*n* = 13, 13.5%) and post-traumatic stress disorder (*n* = 13, 13,5%) were the most common comorbidities. All patients were on oral psychopharmacotherapy at the initiation of esketamine. The mean number of different agents was 3.1 (median 3.0, range 1–6). About 40.6% (*n* = 39) had a history of electroconvulsive therapy (ECT) non-response in the current depressive episode (ECT+), whereas 59.4% (*n* = 57) patients did not receive an adequate course of ECT in their current episode (ECT-). Patients were severely affected, mean MADRS score at baseline was 32.6 (median 32.0) and mean BDI was 37.6 (median 37.0) indicating severe depression. A comparison of baseline disease and treatment characteristics between the ECT + group (history of ECT non-response in the current episode) and the ECT- group is provided in Table 1.

**Table 1:** Comparison of baseline disease and treatment characteristics between patients with (ECT+) history of electroconvulsive therapy (ECT) in the current episode and patients who have not received an adequate course of ECT (ECT-) in the current episode. SSRI: selective serotonin reuptake inhibitor; SNRI: serotonin norepinephrine reuptake inhibitor

		ECT+ (*n* = 39)	ECT- (*n* = 57)	*p* value
Age (mean)		47.3	46.7	0.843
Gender (women, %)		46.2	56.1	0.407
Number of esketamine sessions (mean)		10.6	10.7	0.905
Main indication for esketamine (psychiatric emergency, %)		17.9	15.8	0.787
At least one psychiatric comorbidity (%)		56.4	59.6	0.834
Comorbid medical condition (%)		48.7	50.9	1.000
Mean number of psychotropic agents		3.2	3.0	0.331
Class of antidepressant medication (%)	SSRI SNRI Other antidepressant	7.7 74.4 17.9	28.1 61.4 10.5	0.041

### Treatment course and adverse events

During the first esketamine treatment session, patients received a starting dose of either 56 mg (*n* = 69, 71.9%), 84 mg (*n* = 26, 27.1%) or 28 mg (*n* = 1, 1.0%) esketamine. Among those patients receiving 28 or 56 mg esketamine, the dose has been increased to 84 mg in the majority of cases (*n* = 66, 94,3%) in the subsequent sessions. The dose increase has been realized in the third session (mean 3.0) session. The mean number of esketamine sessions per patient was 10.7 (median 10.5, range 1–25). Treatment discontinuation (5 or less treatment sessions) occurred in 9.4% (*n* = 9) of patients due to intolerable dissociations (*n* = 4), sedation and dizziness (*n* = 1), discontinuation against medical advice (*n* = 2), switch to ECT due to worsening of depression (*n* = 1) and discontinuation due to subjective inefficiency of treatment (*n* = 1). There was one suicide attempt in the whole sample during the esketamine treatment series, no deaths were recorded.

### Effect of disease and treatment-related factors on treatment outcome

#### MADRS

In the factorial analysis of variance (ANOVA), there was a significant interaction effect of time on MADRS score (F = 27.389, *p* < 0.001) indicating a significant alleviation in depression severity from baseline to end of esketamine treatment series in the whole group. There was no significant interaction effect of time*history of ECT non-response in current episode (F = 2.166, *p* = 0.143). The latter did not significantly impact the change in MADRS score from baseline to end of treatment series. However, history of ECT non-response had a significant main effect on MADRS score in the ANOVA with higher overall MADRS scores in ECT non-responders (F = 10.386, *p* = 0.002). The ECT + and ECT- group did not differ regarding the MADRS score at baseline (mean difference 2.6, *p* = 0.208). However, at the end of esketamine treatment the difference between the groups was significant (6.9, *p* = 0.001). MADRS scores were also higher in women than men (F = 6.639, *p* = 0.011) as well as in patients receiving esketamine for the treatment of a psychiatric emergency than in patients treated with esketamine due to TRD without a psychiatric emergency (F = 11.627, *p* < 0.001). None of other variables with fixed effects (psychiatric comorbidity, class of antidepressant medication, gender, indication for esketamine treatment) had a significant interaction effect on change in MADRS score over time (Table 2). Patients with a history of ECT non-response in the current episode (ECT+) experienced significant alleviation of depression severity in the MADRS score. Table 3 provides the mean values and changes from pre-treatment to post-treatment for the different measures including 95%-confidence intervals in the two groups.

**Table 2:** Factorial ANOVA with MADRS score as the dependent variable, fixed factors as independent variables (disease and treatment-related parameters) and age and number of treatment sessions as covariates. None of the variables with fixed effects had a significant interaction effect on change in MADRS score over time

Fixed factor	Main effects		Effect of the interaction time*respective fixed factor	
	F	*p*-value		
Time (pre- /post-treatment)	27.389	< 0.001	F	p-value
ECT non-response in current episode	10.386	0.002	2.166	0.143
Psychiatric comorbidity	0.081	0.776	0.473	0.493
Class of concomitant medication	1.556	0.214	0.755	0.472
Gender	6.639	0.011	1.434	0.233
Indication	11.627	< 0.001	0.013	0.909

**Table 3:** Patients with a history of ECT non-response in the current episode (ECT+) experienced significant alleviation of depression severity in MADRS, in suicidal ideations as well as in the BDI-II score. Since we did not see any significant interaction effect of time*score in any of the rating scales (MADRS, BDI-II or MADRS item-10), the observed differences in mean reduction between the ECT- and the ECT + group were not statistically significant. 95%-CI: 95% confidence interval

		Pre-treatment mean MADRS (95%-CI)	Post-treatment mean MADRS (95%-CI)	Mean Reduction	*p*-value
MADRS	ECT+	37.3 (33.6–41.1)	28.6 (24.9–32.4)	8.7	0.001
	ECT-	34.7 (31.5–38.0)	21.7 (18.3–25.2)	13.0	< 0.001
Suicidality (MADRS item 10)	ECT+	3.5 (3.0-4.1)	2.1 (1.5–2.7)	1.4	< 0.001
	ECT-	2.8 (2.3–3.3)	1.7 (1.2–2.2)	1.1	0.002
BDI-II	ECT+	41.9 (37.2–46.7)	33.1 (28.3–37.8)	8.8	0.010
	ECT-	38.6 (34.5–42.8)	24.0 (19.7–28.3)	14.6	< 0.001

#### Suicidality (MADRS item 10)

There was a significant impact of time on the suicidality score indicating significant reduction of suicidal ideations from baseline to end of esketamine treatment series (F = 17.196, F < 0.001). History of ECT non-response in the current episode was significantly associated with higher overall suicidality scores (F = 6.813, *p* = 0.010). The difference between ECT + and ECT- regarding suicidality was statistically significant at baseline (0.7, *p* = 0.017), whereas the difference at the follow-up was not statistically significant (0.4, *p* = 0.199). However, there was no interaction effect of time*history of ECT non-response on the change in suicidality in the treatment course (F = 0.557, *p* = 0.457). History of ECT non-response did not significantly influence the change in suicidality during esketamine treatment series.

#### BDI-II

**Fig. 1:**
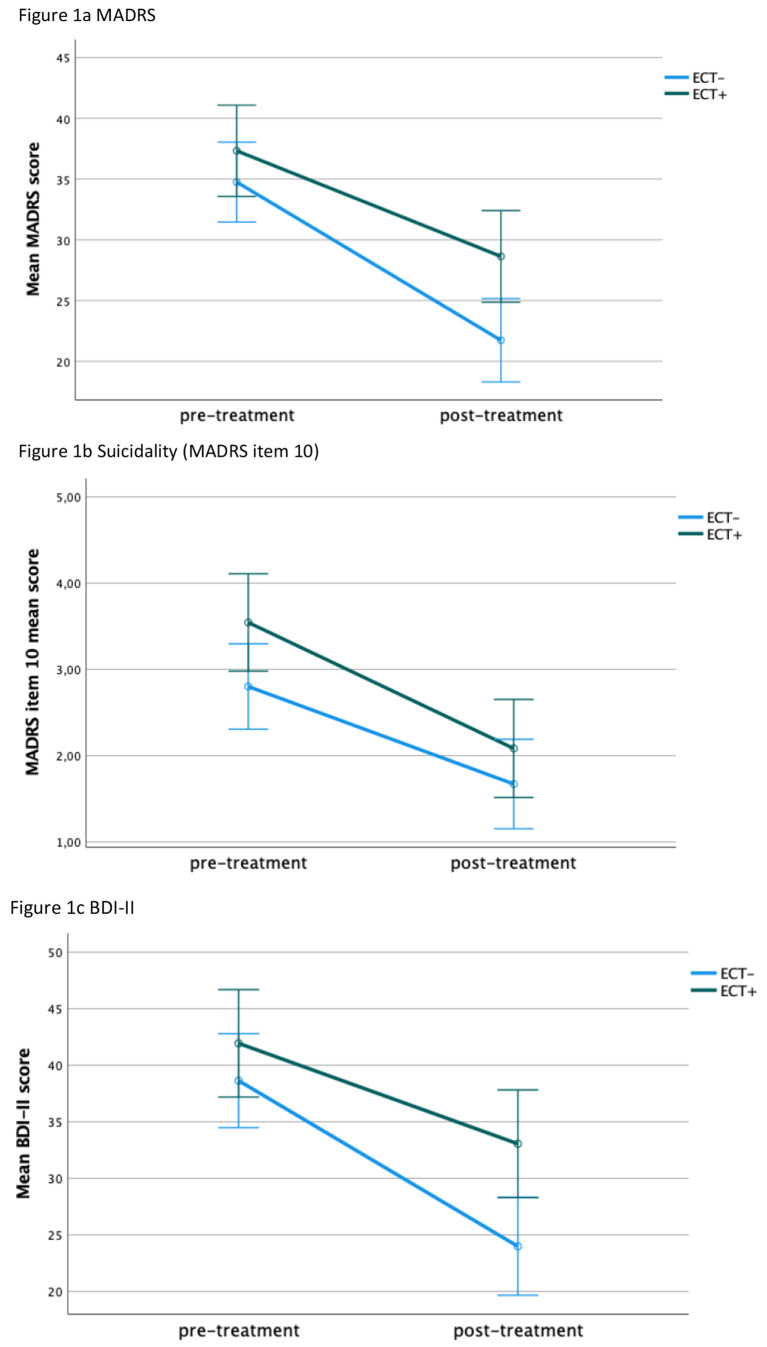
Changes in rating scales from pre-treatment to post-treatment in patients with (ECT+) history of ECT non-response in the current depressive episode and patients who have not received an adequate course of ECT (ECT-)in their current episode. Bars indicate 95%-confidence intervals

The self-rated depression severity in BDI-II also showed a significant interaction effect with time indicating significant overall improvement from baseline to end of treatment series (F = 20.227, *p* < 0.001). Again, history of ECT non-response was associated with significantly higher disease severity (F = 11.045, *p* = 0.001). While the difference in BDI-II between the ECT + and ECT- group was not statistically significant (mean difference 3.3, *p* = 0.203) in the pairwise comparison at baseline, the BDI-II scores significantly differed between these groups after the treatment series (9.1, *p* < 0.001). However, history of ECT non-response did not impact the change in BDI-II over time (F = 2.396, *p* = 0.124). Women as well as patients receiving esketamine due to a psychiatric emergency had significantly higher scores in BDI-II, but these factors also had no impact on BDI-II changes over time. Patients with a history of ECT non-response in the current episode (ECT+) also achieved significant alleviation of depression severity in the BDI-II score. Table 3; Fig. 1 provide information on changes in different outcome measures in both, the ECT- and the ECT + groups.

### Comparison of response and remission rates

The observed differences between the ECT + and the ECT- group regarding response and remission rates were not statistically significant. While 34.9% of the patients responded to esketamine in the ECT- group, the response rate was 21.9% in the ECT + group (χ2 = 1.498, *p* = 0.167). About 12.1% of ECT non-responders achieved remission, while in the ECT- group 24.4% fulfilled remission criteria (χ2 = 1.861, *p* = 0.141).

## Discussion

This study represents the first real-world single-center cohort to include a significant number of patients with a history of ECT non-response in the current depressive episode. Overall, our study revealed significant alleviation of depression severity as well as reduction in suicidal ideations following esketamine nasal spray treatment in highly affected individuals, who had been hospitalized due to treatment-resistant depression. Our data confirm the efficacy of esketamine intranasal spray in a real-world setting. The seen improvement in depression severity occurred in both the investigator rated as well as the patient rated depression measures.

Our data indicate that patients with a history of electroconvulsive therapy non-response in the current major depressive episode are more severely affected. Although, the observed effects on the change in depression severity following esketamine treatment tended to be less pronounced in previous ECT non-responders, the differences were not statistically significant. Overall, there was no statistically significant negative impact of ECT non-response in the current episode on the change in depression severity nor on response and remission rates following esketamine treatment.

To the best of our knowledge, this is the largest report on the outcome of esketamine in ECT non-responders. Patients with a history of electroconvulsive therapy non-response usually have been excluded from participation in the TRANSFORM-1 and TRANSFORM-2 studies [6, 7], which assessed the acute efficacy of esketamine intranasal spray. In the ESCAPE-TRD trial comparing esketamine vs. quetiapine in patients receiving an SSRI or SNRI, ECT non-response was also an exclusion criterion [13]. Thus, there is lack of data regarding the efficacy of esketamine in ECT non-responders. Martinolli et al. included only one patient with a history of ECT [14]. In a French real-world sample, about 42% of the patients have been treated with ECT prior to esketamine, about 39% were resistant to ECT [19]. However, the authors did not report on the impact of ECT resistance on esketamine treatment outcome [19]. In a small case series, 6 out of 16 patients had a history of ECT [20]. In that study, history of ECT did not affect the outcome of esketamine nasal spray treatment, which is in line with our results. However, the authors did not report on the impact of ECT response status on esketamine treatment outcome [20]. Our results indicate that, in a naturalistic design, esketamine nasal spray can provide clinical improvement in individuals who previously did not respond to ECT in their current depressive episode. We observed that treatment effects tended to be smaller in previous ECT non-responders. However, since the results were not statistically significant, our results do not confirm the assumption that previous ECT non-response has a negative impact on esketamine treatment outcome. Additionally, the study may have been underpowered to detect between-group effects and a possible negative impact of ECT non-response on esketamine treatment outcome. Therefore, whether ECT non-response has a negative impact on esketamin treatment outcome or not should be investigated in future prospective, randomized controlled trials.

Since ECT non-responders benefited from esketamine treatment, our results support the approach to offer esketamine to those individuals with a history of ECT non-response in the current episode, particularly given that the array of treatment alternatives is limited after an unsuccessful course of ECT. Intermittent theta burst (iTBS) transcranial magnetic stimulation could also be a treatment option in patients after an unsuccessful series of ECT. Data on the outcome of ITBS in ECT non-responders are sparse. We recently reported that the probability of response to iTBS decreases dramatically to 10% in case of ECT non-response in the current episode [21]. Keeping in mind the limitations of the retrospective, non-randomized, naturalistic and unblinded study design, our results do no allow any comparison on the superiority of either esketamine or iTBS in previous ECT non-responders. Future head-to-head comparisons may answer this question, which would be a useful contribution to existing literature, when establishing a treatment algorithm for neuromodulatory treatments in major depression.

Overall, no life-threatening adverse events occurred during esketamine treatment. We observed one suicide attempt, which did not result in death. The number of treatment discontinuations (9.4%) was similar to other reports [7, 14]. Thus, esketamine treatment can be considered as a safe procedure.

In summary, we observed significant improvement of depression symptoms as well as suicidal ideations in highly affected inpatients with treatment-resistant depression and a history of ECT non-response in a real-world setting. No safety concerns became apparent, confirming the safety of esketamine intranasal treatment for real-world use.

### Strengths and limitations

This study is the largest study to report on the efficacy of esketamine in patients with a history of ECT non-response in the current episode, which has been an exclusion criterion in previous esketamine trials. It is also one of the largest single center real-world reports confirming the efficacy and safety of intranasal esketamine in real-world clinical practice. However, the study may have been underpowered to detected between-group effects of ECT pretreatment on treatment outcomes with esketamine.

Our results also demonstrate the safety and efficacy in a setting of patients with high prevalent psychiatric comorbidities. However, the main limitation of the study is the naturalistic and retrospective design with no randomization and no control group. Furthermore, patients also received standard of care treatment (psychotherapy, oral medication) in addition to esketamine. The ratings took place in routine clinical practice, thus the raters were not blinded. Furthermore, we did not use a dedicated suicidality scale. Instead, the suicidality item (item 10) of the MADRS was used to rate suicidality. The use of the suicidality item of the depression rating scale may have caused a parallelization in the rating of depression severity and suicidality. Another limitation of our study is that we did not collect data on the course of the previous ECT series. More detailed information on the course of ECT and the ECT technique, including electrode placement and number of sessions, would have been helpful.

Future studies should focus on patients with psychiatric comorbidities as well as patients with history of non-response to other neuromodulatory techniques. The management of esketamine non-response may be a focus of future clinical research.
